# Cytomegalovirus latency—the sum of subtleties

**DOI:** 10.1128/jvi.00664-25

**Published:** 2025-07-30

**Authors:** Meaghan H. Hancock, Patrizia Caposio, Donna Collins-McMillen, Nicole L. Diggins, Byeong-Jae Lee, Samuel Medica, Daniel N. Streblow, Timothy White, Andrew D. Yurochko, Felicia Goodrum

**Affiliations:** 1 Vaccine and Gene Therapy Institute, Oregon Health and Science Institute 828705, Beaverton, Oregon, USA; 2 Department of Microbiology and Immunology, Geisel School of Medicine at Dartmouth, Lebanon, New Hampshire, USA; 3 Department of Microbiology and Immunology, Louisiana State University Health Sciences Center 547965, Shreveport, Louisiana, USA; Universiteit Gent, Merelbeke, Belgium

**Keywords:** virus-host interactions, cytomegalovirus, latency, herpesvirus

## Abstract

Human cytomegalovirus (HCMV) is a betaherpesvirus, which, like all herpesviruses, establishes a life-long latent infection while retaining the ability to reactivate its replicative program. While HCMV likely reactivates frequently and sporadically in healthy individuals and typically without disease, reactivation poses a serious disease threat in the immunocompromised. The latent program of HCMV is complex and has been challenging to define due to limitations in appropriate experimental model systems related to virus-host species specificity, limited identification of *in vivo* latent reservoirs, and the dynamic cellular differentiation of the hematopoietic latency reservoir that is directly linked to latency maintenance and reactivation phenotypes. Here, we review the current understanding of HCMV latency, with a focus on cross-cutting principles derived collectively from *in vitro* experimental culture models and *in vivo* animal models using the corresponding orthologs (CMVs) to HCMV.

## INTRODUCTION

Herpesvirus infections are characterized by life-long infections due to the establishment of latency, with repeated bouts of reactivation that ultimately maintain infection in the host and provide the potential to cause disease and/or transmit to a new host. Since the early 1920s, we have appreciated that a non-replicative conserved form of herpesviruses following primary infection is maintained and can cause recurrent disease, although it took another 50 years to gain insight into the cell types involved and the mechanisms that govern latency (1). Decades of work in herpes virology, particularly herpes simplex virus (HSV) and Epstein-Barr virus (EBV), have defined latency as a state in which viral genomes are maintained, genes important for replication are largely silenced while genes important for latency are more highly expressed, and infectious progeny are not detected (2). The virus episodically emerges from latency to replicate, with the potential to cause disease and spread in an event termed reactivation. While latency is primarily characterized by a repression or change in viral transcription due to chromatin modification, infection events driving silencing or de-repression that underlie latent or replicative programs, respectively, are influenced strongly by the cell type infected, its profile of gene expression (e.g., available transcription factors or other host factors) at the time of infection, and its intrinsic and innate responses that can vary significantly cell-to-cell and among cell types or differentiation stages. These factors influence the pattern of viral gene expression by influencing chromatinization of the genome and the resulting relative abundance of immediate early transcripts encoding proteins and non-coding RNAs required for replication. If thresholds of viral gene expression required for replication are not reached due to restrictive host-virus interactions, then a latent infection may be established or maintained.

HSV-1 and -2 establish latency in terminally differentiated peripheral neurons in sensory and autonomic ganglia and are defined by the expression of latency-associated transcripts and microRNAs (miRNAs) from the same locus in the absence of comparable expression from other viral genes. EBV latency is defined by sets of unique gene expression programs regulated by B-cell differentiation that drive latency and contribute to tumorigenesis. Furthermore, while HSV-1 is not thought to require mechanisms to maintain its genome in non-dividing neurons, EBV actively tethers its episomal genome to cellular chromatin. Human cytomegalovirus (HCMV), the prototypical beta herpesvirus and the largest member of the *Herpesviridae* family, establishes life-long infections following acute infection but targets different primary cell types and, while similarities exist in some host pathways important for latency, divergent mechanisms for viral maintenance and reactivation also exist compared to other herpesviruses. Defining the HCMV latency program(s) has been challenging due to the large genome capacity of HCMV, encoding well over 200 proteins and non-coding RNAs, and the exceptionally broad tropism of virus for primary human cells. A restricted program of gene expression characteristic of alpha- and gamma-herpesvirus latent infection has yet to be defined. Indeed, recent studies using state-of-the-art highly sensitive sequencing approaches detect broad, albeit low-level, viral gene expression from across the HCMV genome (3–6). While we and others have defined several HCMV genes and miRNAs as contributors to latency and/or reactivation, the expression of these factors is not strictly restricted to “latently” infected cells. Furthermore, as the sensitivity of methods to detect gene expression in rare “latently infected cells” increases, the periods where viral gene expression and infectious virus cannot be detected between episodes of “reactivation” have decreased. How CMVs maintain their genomes is also not clear. While a chromatin tethering domain has been characterized in major and minor IE1 proteins that is important for binding histones and maintaining genomes through S phase (7, 8) and interacts with host topoisomerase II beta (9), the role of this activity for genome maintenance in latency requires further study. Over our collective years of studying latency in a variety of human and animal models, we are left to ask whether CMV latency is wholescale distinct from that defined by other *Herpesviridae* members, whether we have yet to define the precise reservoir for latency in representative model systems, or whether it is the definition of latency itself that needs refinement, at least in the case of CMVs.

Our goal here is to provide an overview of the state-of-the-art pertaining to CMV latency, considering the existing body of literature from clinical and basic science studies in animal and *in vitro* culture models. We turn a critical eye to the limitations of experimental and non-human animal model systems, the general principles cutting across systems, and how they inform what we understand about CMV latency, and what we still do not know.

## THE PROBLEM OF HCMV LATENCY

Understanding latency requires differentiating it from persistence. Persistent infections are those in which low levels of infectious virus can be detected in the host for long periods. By contrast, latent infections are those where the infectious virus cannot be detected between episodes of acute viral shedding. Within the organism, low-level, asymptomatic persistent virus shedding is frequently detected for most herpesviruses (10–12), suggesting that true latency on the organismal level is transient at best, even in the absence of symptoms or disease. The goal then must be to understand infection in cell types that contribute to the persistence of the virus, those where virus replication is entirely (latent) or strongly restricted to escape detection and maintain infection, and how these infections differ from cells productively infected.

**Fig 1:**
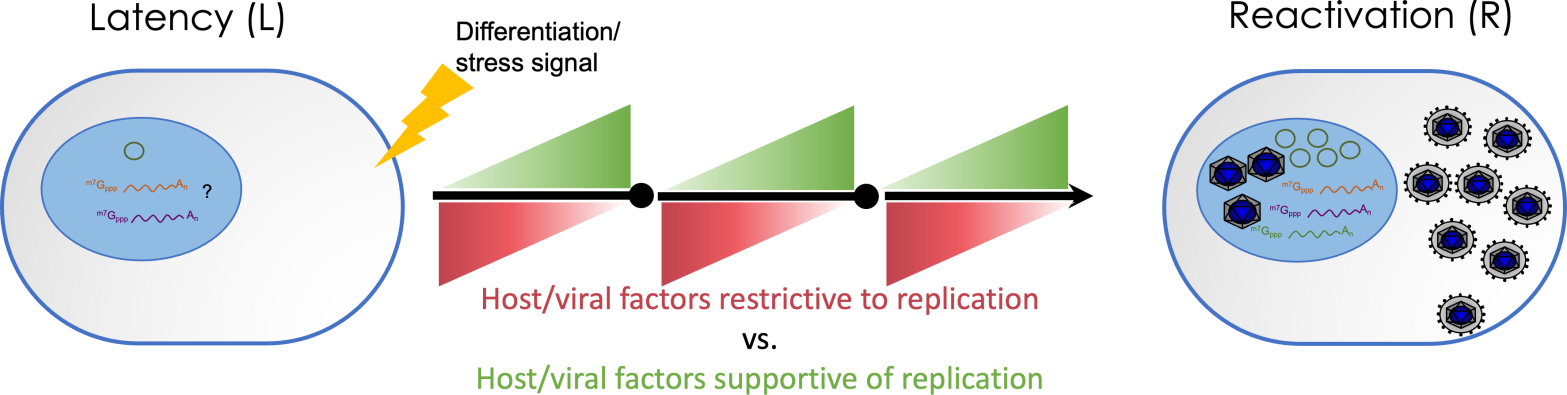
Schematic of latency and reactivation. The nature of the latent infection in HCMV remains elusive. However, our mechanistic insights have grown dramatically over the past two decades due to innovation in experimental models, *in vitro* and *in vivo*, and viral genetics relying on bacterial artificial chromosome clones of HCMV. The establishment of latency or reactivation from latency depends on the biology of the infected cell and a multitude of interconnecting virus-host interactions (Fig. 2) that remain to be completely defined. A central theme emerging from research carried out by many groups is that latency is established by host restrictions that reversibly restrict viral gene expression without aborting infection following virus entry. Reactivation is stimulated through differentiation- or stress-induced reduction in host restrictions (red) coupled with increases in viral gene expression and host factors (green) that accumulate to thresholds to cross checkpoints for commitment to virus reactivation. The virus has evolved to sense and respond to changes in host signaling, as evidenced by host transcription factor control of virus gene expression, susceptibility of viral factors to selective host degradation, and a plethora of virus-host interactions to tweak biology of the infected cell. Fundamental questions remain to be fully defined, including: (i) how is the viral genome silenced and maintained, (ii) what is the latent transcriptome, (iii) what are the additional reservoirs of HCMV latency, and (iv) how are diverse signals integrated to make decisions around the maintenance of or reactivation from latency?

The premise of a latent state is the restriction of viral gene expression to prevent immune detection and production of infectious virus. Following from this, latency has sometimes been thought of in terms of on or off states: genes important for productive replication must be off while genes important for latency must be on. However, a restricted viral transcriptome unique to latency has been elusive even in homogeneous cell lines (13) or using single-cell approaches (4). Work over the past several decades demonstrates that CMV latency culminates from a complex network of subtle interactions between viral and cellular factors (e.g., proteins, micro- and long non-coding RNAs, miRNAs, and lncRNAs, respectively). These are regulated by a multitude of subtle pushes and pulls that together shape the outcome of virus infection. We are beginning to appreciate how miRNAs and proteins work together to collectively change the environment of the cell for latency or replication. Some viral genes, such as the US28 G protein-coupled receptor (GPCR), which binds several chemokines from different families resulting in distinct impacts on downstream signaling cascades, have roles in both latency and reactivation. Finally, given the preponderance of host transcription factors controlling HCMV gene expression, especially that of key genes such as the major immediate early transactivators, we propose that latency is dictated primarily by the profile of host factors available to restrict or support virus replication in the infected cell. A network of virus-host interactions is emerging, where thresholds of host or viral factors need to be reached to clear checkpoints that regulate viral gene expression and the ability of the infection to progress or not—ultimately a replicative or latent outcome (Fig. 1). In such a system, even broad viral gene expression may not reach levels sufficient for commitment to synthesis of viral DNA or production of progeny.

## CD34^+^ HEMATOPOIETIC PROGENITOR CELLS (HPCS) AS A RESERVOIR FOR LATENT HCMV

A hematopoietic reservoir for latent virus was initially postulated based on evidence that infectious virus-free leukocytes could transmit HCMV infection (14). Studies have primarily focused on CD34^+^ HPCs and other cells of the myeloid lineage, including monocytes, based on the detection of viral genomes in these cells in healthy seropositive individuals and the ability to stimulate reactivation *ex vivo* from these cell types (15–18). Moreover, transplantation of G-CSF mobilized CD34^+^ peripheral blood stem cells from seropositive donors can result in virus transmission to seronegative recipients (19). There are undoubtedly important differences in HCMV latency programs that remain to be systematically understood between progenitor cells, monocytes, and other possible hematopoietic reservoirs for latency. CD34^+^ HPCs are a well-regarded model for HCMV latency and reactivation among *in vitro* human experimental systems. CD34^+^ HPCs are a heterogeneous population of cells that exist along a spectrum from early pluripotent progenitors to those at the point of lineage commitment. CD34^+^ HPCs are isolated from bone marrow, cord blood, or fetal liver with a high degree of purity and are susceptible to HCMV infection. Studies utilizing primary CD34^+^ HPCs have increased our knowledge of the molecular mechanisms involved in latency and reactivation and have identified roles for HCMV genes that do not have homologs in murine CMV (MCMV) or rat CMV (RCMV) (18, 20–34).

**Fig 2:**
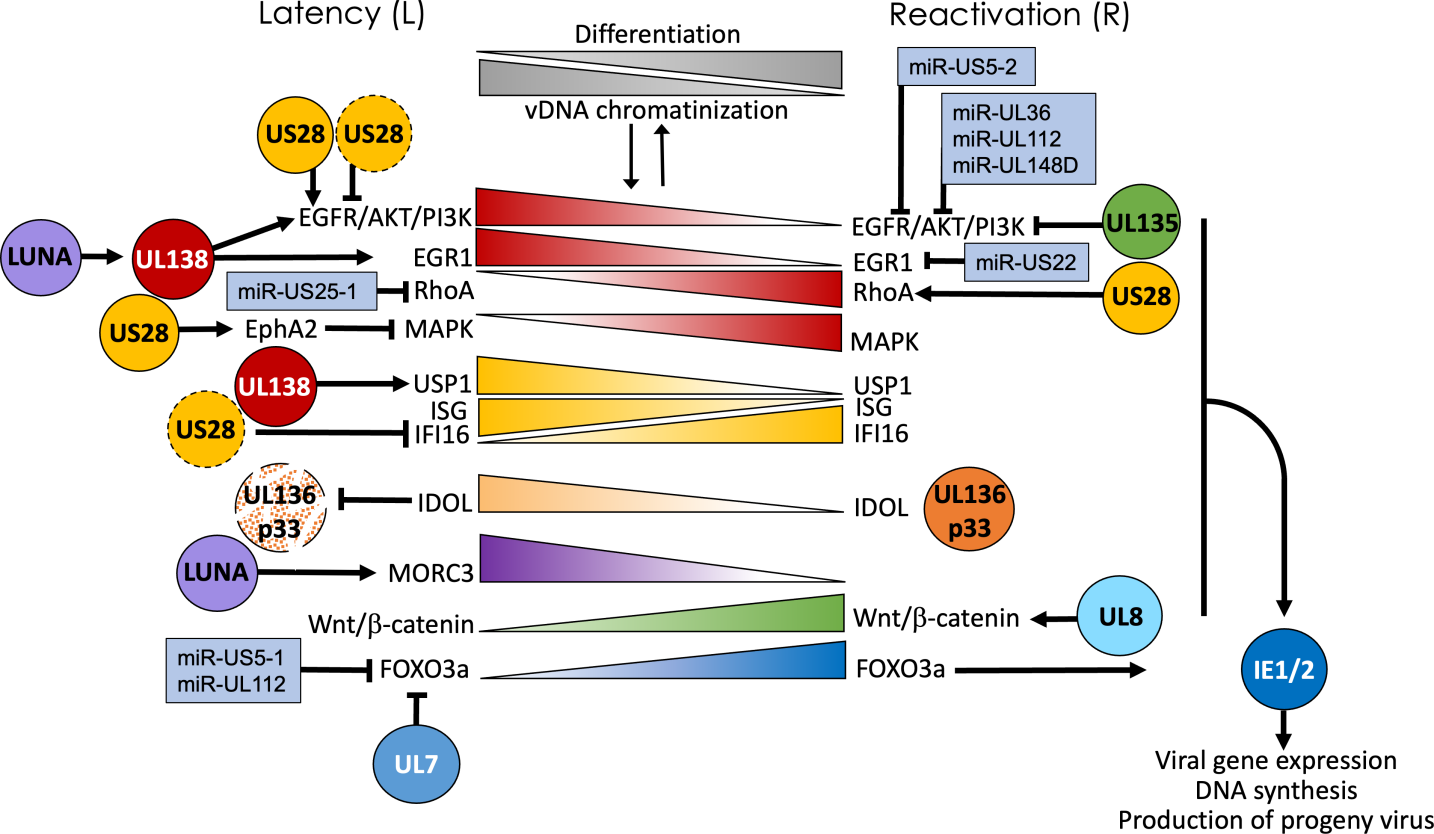
Hematopoietic differentiation-associated host-virus interactions regulating latency and reactivation. HCMV latency and reactivation are tied to hematopoietic differentiation and corresponding changes in chromatinization of the viral genomes and patterns of viral gene expression (gray gradients). HCMV latency and reactivation decisions are modulated by the integration of diverse cellular information through a series of complex virus-host interactions, which are remarkably defined by hematopoietic differentiation. While this figure is not intended to be comprehensive, we represent key host pathways with gradients to reflect their general regulation through hematopoietic differentiation. Viral proteins (spheres) and miRNAs (blue boxes) and their proposed modulation of these pathways and impact infection outcomes—latency or reactivation—are shown. Some of these interactions are known to influence hematopoietic differentiation, particularly in the case of US28 and viral miRNAs, which target major signaling pathways important to stress responses and cellular differentiation. The EGFR→AKT→PI3K and related-Rho and MAPK signaling axis is important in regulating latency/reactivation decisions (red gradients). These pathways are regulated by US28, UL138, and UL135, as well as v-miRNAs. Type-1 IFN signaling and expression of ISGs are also thought to promote a latent state and can be regulated by US28 and UL138. In the case of UL136, a determinant important for reactivation, its levels are regulated by the IDOL host E3 ubiquitin ligase, which is regulated by LXR signaling through differentiation (orange gradient). High levels of IDOL present in undifferentiated hematopoietic cells maintain low levels of UL136p33 for latency, and loss of IDOL with differentiation allows UL136p33 to accumulate and drive increased IE gene expression and virus reactivation. LUNA desumoylates MORC3 (purple gradient), which represses IE and stimulates UL138 gene expression (46, 47). UL8 interacts and stabilizes components of the Wnt/β-catenin pathway for reactivation (green gradient). Finally, expression of the host transcription factor FOXO3 is increased with differentiation (blue gradient) and is important for activation of alternative intronic promoters regulating IE expression. FOXO3 is maintained at low levels during latency by UL7 and v-miRNAs. Most interactions on the side of reactivation directly or indirectly contribute to increased activation of IE genes. Much remains to be understood about the coordination of and interplay between these host pathways and viral regulators. US28 is particularly complex, and some phenotypes have been defined in CD34+ cells (solid sphere outline) or monocytes (indicated by dashed sphere outline), which we have tried to differentiate.

In one hematopoietic long-term culture model (35), latently infected cells are cultured over stromal cells designed to maintain the “stemness” of hematopoietic progenitor populations to the extent that they can be used to reconstitute the hematopoietic compartments of sub-lethally irradiated mice (36, 37). The stromal cell support is important for maintaining cells in a state that will support latency by limiting spurious differentiation. In this culture system, HCMV infection of CD34^+^ cells is characterized by very low levels of viral transcripts (5, 38, 39) compared to replication-permissive cell types. Detection of viral genomes decreases over time, and the number of progeny virus produced when cells are stimulated to reactivate is very low (~1 in 9,000 cells) (39). It is not clear whether the decrease in viral genomes is a caveat of the culture system or part of the biology of HCMV latency in this cell type. However, it is worth noting that loss of genomes appears to be a consequence of cellular division, as genomes are maintained in non-dividing primary monocytes while the number of genomes per cell decreases over time in proliferating THP-1 cells (40, 41). Likewise, as CD34^+^ HPCs continue to divide following infection, it is currently unknown whether genomes are diluted out by cell division or if only a small subset of the cells initially infected can maintain genomes. Low levels of infectious virus produced during long-term culture likely result from spontaneous differentiation occurring in a small fraction of cells. To stimulate reactivation, infected cells are co-cultured with fibroblasts using a cytokine cocktail to stimulate differentiation, leading to the production of viral progeny. Reactivation is performed at 10–14 dpi with frequencies of reactivation diminishing beyond that. The CD34^+^ HPC long-term culture model has been used to identify viral genes and associated host pathways that function to restrict or amplify virus replication in hematopoietic cells, and thus, is interpreted to function in the establishment of latency or reactivation. Many of the host factors important to latency and reactivation are regulated through hematopoietic differentiation (e.g., highly expressed in latency and downregulated by differentiation or vice versa) (Fig. 2) (42–45). Importantly, many of the latency and reactivation phenotypes identified in CD34^+^ HPCs have been recapitulated in the humanized mouse model described below, thus validating this *in vitro* model as physiologically relevant to viral infection *in vivo*.

The advent of widely available high-throughput sequencing technologies allowed complete transcriptomes of HCMV infection to paint a much different picture of latency than originally defined, with a broader and dynamic gene expression pattern than previously imagined (27, 39, 48, 49). As a pooled population, latently infected CD34^+^ cells transcribe the full complement of genes expressed during lytic infection, although at much lower levels (4, 5, 13). These studies were able to detect differences in both comparative expression levels and comparative kinetics of individual viral genes across time points, suggesting some level of viral gene regulation in CD34^+^ HPCs. An analysis of the latent transcriptome using single-cell sequencing of CD14^+^ monocytes and CD34^+^ progenitor cells revealed an array of transcriptional profiles, from broad to narrow, distributed throughout heterogeneous populations (4). Although gene expression is restricted during latency, studies to date suggest that the latent infection is not transcriptionally silent, nor restricted to a few “latency” genes, and that there is no binary “on/off” switch to control viral gene expression.

Limitations of the CD34^+^ HPC model include the limited quantities and expense of the collection of CD34^+^ cells from human donors, preferably from bone marrow or cord blood. While mobilized peripheral blood stem cells are more readily available, G-CSF treatment alters the activation and differentiation state of CD34^+^ HPCs and has been shown to mobilize myeloid cells and trigger HCMV reactivation in latently infected humanized mice (50). Although mobilized PBSCs have been used in some latency models (19, 51, 52), we and others have found that they may be too stimulated to reliably support latency relative to CD34^+^ cells derived from bone marrow or cord blood (19, 53). Fetal liver is also a rich source of CD34^+^ HPCs and is ideal for the reconstitution of humanized mice, but it is not broadly available and differs phenotypically from adult human bone marrow-derived cells. Notably, fetal CD34^+^ HPCs exhibit enhanced proliferative potential, a bias toward erythroid and megakaryocytic lineages, and lower expression of key surface markers involved in immune cell maturation (54, 55). The CD34^+^ HPC infection model also has inherent donor-to-donor variability that can affect reproducibility and complicate statistical analyses. In addition, the natural heterogeneity of the CD34^+^ population and the varied outcome of infection among the different subpopulations (48, 56) create variability between results with different donors. It is expected that these limitations will be addressed with the increasing availability of single-cell technologies. Finally, while technology to knock down or manipulate host gene expression or overexpress a transgene in CD34^+^ cells exists, this remains a significant challenge in the context of HCMV infection. Genetic manipulation (e.g., lentiviral transduction or nanoparticle electroporation) and subsequent selection alter the biology of the cells in ways that affect the outcome of infection. Further compounding this issue, the low efficiency of these techniques, coupled with that of subsequent infection, has resulted in cell numbers restrictive to most downstream analysis.

To address some of these limitations, L. B. Crawford et al. (56) developed a CD34^+^ HPC model derived from human embryonic stem cells (hESCs) that recapitulates many of the hallmarks of the donor-derived CD34^+^ HPC *in vitro* system (25, 35, 39). hESC-derived CD34^+^ HPCs support latency and virus reactivation (56), have been used to define roles for viral proteins and miRNAs in latency (33, 57, 58), and addressed heterogeneity in infection outcomes in hematopoietic subpopulations (56). hESC-derived CD34^+^ HPCs offer several benefits over primary CD34^+^ HPC culture. WA01 and WA09 hESC lines are pluripotent cells that have a normal karyotype and high telomerase activity so that they can be banked and cultured in an undifferentiated state indefinitely (59), eliminating the issue of donor variability. The use of commercial kits to differentiate hESCs into CD34^+^ HPCs also enhances the reproducibility of experiments through the utilization of a defined culture medium. Moreover, unlike donor sources, expansion of hESCs prior to differentiation provides a system that is scalable, allowing for analyses that require greater levels of starting material, which was previously not feasible using primary HPCs without pooling cells from multiple donors. Lastly, the frequency of cells capable of reactivation is higher in hESC-derived cells (~1 in 300 cells reactivates to form an infectious center) (56) compared to primary cells where reactivation is a rare event (~1 in 9,000 cells) (60), thus allowing a greater dynamic range of reactivation capacity and the ability to capture and quantify intermediate phenotypes.

## CD14^+^ MONOCYTES CARRY LATENT HCMV

Monocytes are essential for hematogenous dissemination of HCMV following primary infection and the establishment of lifelong infection of the host (reviewed in references [61–69]). Monocytes likely acquire virus from epithelial cells at the site of initial infection (analogous to EBV infection of epithelial cells and B cells) and traffic to the bone marrow, where they transfer infection to CD34^+^ HPCs, although this remains to be definitively shown. Infected monocytes upregulate adhesion receptors and/or their function and the ability to attach to the endothelium, as well as acquire motile features (actin cytoskeletal changes, cellular activation) to extravasate to organ tissue from the blood (reviewed in references [62, 63, 65, 68–73]). Moreover, differentiation of infected CD34^+^ HPCs toward cells of the monocytes/macrophage lineage results in reactivation (50), and HCMV utilizes the immunological features of monocytes/macrophages (reviewed in references [53, 74]) to promote viral spread from the blood and persistence in organ tissue as long-lived tissue macrophages.

Monocytes are highly plastic cells and not a single cell type, but can be loosely grouped into different subpopulations (classical, intermediate, and nonclassical monocytes based on CD14 and CD16 surface marker expression) with different reported functions (53, 74). Within defined monocyte and macrophage subpopulations, there also likely exists a gradient of different cell types with subtle differences in biological functions (53, 74). Whether the virus infects a distinct subpopulation or drives differentiation of a distinct population of macrophages remains to be completely defined. It remains unclear whether HCMV reactivation promotes or requires a specific monocyte or macrophage subpopulation. Phenotypic and transcriptomic analyses suggest that infected monocytes/macrophages are driven toward a biased M1 proinflammatory macrophage-like cell, although additional studies need to refine this process and the nature of this infected cell type (61, 75–77). The process by which the cells are harvested and cultured (78, 79) can affect their phenotype and the outcome of infection.

Following infection of monocytes *in vitro*, translocation of the genome to the nucleus and gene expression have significantly delayed kinetics compared to infection of fibroblasts (80–82). Nuclear translocation of the genome in infected monocytes may require 3 days and virus gene expression is delayed until around 2–3 weeks following differentiation into macrophages (63, 81, 83–85), consistent with *in vivo* observations (63, 83, 85–87), although others have reported earlier gene expression profiles when macrophages are infected (88, 89). HCMV gene products, RNAs, and miRNAs (e.g., US28, UL7, LUNA) regulate viral gene expression and replication in monocytes, monocyte-to-macrophage differentiation, and reactivation from latency (25, 31, 33, 46, 47, 90–94). UL7 is a novel Flt3 receptor ligand that induces myelopoiesis and monocyte to macrophage differentiation and the reactivation process (20). Like UL7, US28 can regulate monocyte to macrophage differentiation and reactivation from latency (25, 93–96). Unsurprisingly, host mitogen-activated protein kinases (MAPKs) and AKT signaling pathways are modulated by viral gene products and impact latency establishment and reactivation (89, 90, 97–100), although there are likely cell-type-specific differences in the regulation of signaling pathways between progenitor cells and monocytes.

Other studies have uncovered key elements about the viral regulation of these myeloid cells from the key viral ligands (i.e., glycoprotein B (gB), the pentamer complex, and others [83, 86, 101]) and the cognate receptors they utilize to attach to the cell (epidermal growth factor receptor [EGFR], integrins, OR14I1, and others [83, 86, 87, 102, 103]). The attachment and entry processes activate a cascade of signaling molecules/pathways through c-Src, EGFR, MAPK, paxillin, phosphoinositol-3 kinase (PI3K), AKT, phosphatase, and tensin homolog (PTEN) (81, 83, 87, 104, 105). Signaling pathways further modulated post-entry (soluble N-ethyl-maleimide-sensitive factor attachment protein receptors [SNAREs], such as syntaxin 6, EGFR, PI3K, AKT, MAPKs and c-Src, PTEN, various antiapoptotic factors (Mcl-1, Bcl-2 family members) [80, 81, 91, 100, 105–111]) alter the expression of transcription factors (e.g., NF-κB, Sp1, AP1, STAT1, c-Fos, and others [77, 112–117]) and this induces long-term changes in these cells (enhanced motility, enhanced survival, and differentiation [63, 65, 86, 118–120]) following infection. These studies have identified key signaling pathways and should be further investigated for their roles in latency and reactivation.

Various human myelomonocytic tumor cell lines have been used in HCMV research, including THP-1, Kasumi-3, HL-60, and U937 cells (13, 29, 38, 92, 121–128). THP-1 and Kasumi-3 (124, 129, 130) are clonal tumor cell lines that represent a developmental precursor of monocytes and can be stimulated to differentiate into macrophages following phorbol ester or inflammatory cytokine treatment. Upon HCMV infection of THP-1 cells, broad gene expression is observed, which is then subsequently largely silenced (13, 38, 131). Differentiation stimulates re-expression of viral genes (13, 38, 131), although THP-1-derived macrophages do not seem to support robust viral genome synthesis or virus production, which is not unlike observations from macrophages derived from CD14+ monocytes (13, 132). The strength of this model, used in many studies, has been in deciphering molecular mechanisms controlling the silencing and re-expression of viral genes that accompany latency and reactivation, respectively. The synchronous silencing and re-expression of viral genes were important for identifying alternative immediate early promoter sequences required for re-expression of IE genes in reactivation (38), a finding validated in CD34+ HPCs. LUNA has been shown to interact with the GATA2 hematopoietic transcription factor in THP-1 cells (46). Furthermore, Kasumi-3 cells have been used to explore roles of viral genes (29) and host signaling pathways in latency (29, 124, 126, 129, 130, 133). While these cell models have served a vital role in understanding HCMV infection and latency in hematopoietic cell models, they have limitations in that their differentiation potential is highly constrained, particularly in the case of THP-1 cells, as they do not support robust viral genome amplification or progeny production following a reactivation stimulus. Thus, it becomes important that observations in cell lines be followed up with studies in primary cells, such as validating data from THP-1 cells in CD14^+^ monocytes (92, 134). There are other cell line models that can be manipulated to study HCMV infection/reactivation/latency. For example, there is also an iPSC model to study myeloid lineage infection (135), as well as a granulocyte-macrophage progenitor model (136).

## THE HUMANIZED MOUSE AS A MODEL FOR HCMV INFECTION *IN VIVO*

The strict species tropism of HCMV has presented significant obstacles in the development of animal models to study HCMV latency and reactivation. As a result, the most suitable *in vivo* model for HCMV involves humanized mice, wherein animals are engrafted with human cells or tissues (137, 138). Since myeloid lineage cells play an integral role in HCMV latency, persistence, and dissemination to organ tissue, Smith et al. developed the first humanized mouse model for HCMV infection in which the mouse bone marrow was engrafted with human CD34^+^ HPCs (50). In this model, adult NOD-scid IL2Rgamma^null^ (NSG) mice were sub-lethally irradiated and engrafted with human cord blood-derived CD34^+^ HPCs to generate humanized NSG (huNSG) mice. At 8 weeks post-engraftment, approximately 5% of peripheral blood mononuclear cells (PBMCs) were human monocytes (huCD45^+^/huCD33^+^/huCD14^+^), similar to the 10% of monocytes found in the PBMC of healthy humans. After intraperitoneal injection of HCMV-infected fibroblasts, HCMV DNA is detected at low levels in organ tissue samples repopulated with human hematopoietic lineage cells. Treatment of the huNSG mice with G-CSF and the CXCR4 inhibitor AMD3100 increases the percentage of human peripheral blood monocytes (to approximately 24%) and mobilizes infected cells into the peripheral blood, spleen, liver, and kidneys of mice. This process results in the expression of early and late HCMV transcripts and proteins in the liver tissue, which exclusively colocalize with cellular markers for human monocytes and macrophages, indicating that HCMV infection occurs solely in the human cells repopulating the huNSG mouse. The huNSG model has also been used to explore HCMV transmission during peripheral blood stem cell transplantation (19). Transplantation of G-CSF-mobilized cells from healthy, HCMV-seropositive human donors into HCMV-negative humanized NSG mice resulted in the detection of viral DNA in the bone marrow, liver, and spleen post-transplantation, indicating successful transmission and dissemination of the virus and supporting the clinical observation that HCMV infection can be transferred by hematopoietic cells in the absence of infectious virus detection.

In the huNSG model, immune responses cannot be measured due to the lack of functional B-cells, CD4^+^ and CD8^+^ T cells, and dendritic cells, and limited reconstitution of endothelial and epithelial cells. This limitation was overcome by humanizing mice with human fetal bone marrow, liver, and thymus tissue (BLT) (139). The huBLT mice exhibit improved systemic reconstitution of human hematopoietic cells, including myeloid lineage cells, NK cells, and CD4^+^ and CD8^+^ T cells due, in part, to the presence of human thymic epithelium. Establishment of latent HCMV infection in huBLT mice results in HCMV-specific human CD4^+^ and CD8^+^ T-cell responses, along with the production of HCMV-neutralizing IgM and IgG antibodies. Furthermore, G-CSF stimulates reactivation and virus spread to peripheral tissues (50, 139). This model offers a valuable platform for investigating HCMV latency and reactivation within the context of a functional immune system, providing insights into how the virus modulates immune responses during these stages of infection.

HCMV humanized mouse models have been crucial for validating the significance of viral factors in latency and reactivation within a living organism. Indeed, this model offers valuable insights into the impact of key host factors and signaling pathways that the virus manipulates to establish a latent infection. In addition, humanized mice have proven beneficial in examining the influence of viral gene products on the maturation of different hematopoietic cell populations (20, 34, 45, 90, 140, 141). While infectious HCMV can be recovered from the spleen and liver in humanized mouse models, reliable quantification of viral load in these tissues is challenging due to the inherent variability in the level of human cell engraftment across individual mice, as well as the heterogeneous ratio of human to mouse cells within any given tissue sample. Furthermore, while the increase in viral genomes within the spleen and liver of infected animals post-reactivation stimulus is interpreted to be the result of enhanced viral replication in differentiated cells, it could also result from a virus-mediated increase in the trafficking of infected cells into the tissues. More detailed immunophenotyping of the cells present in the tissues, looking for changes in the frequencies or activation states of different cell types that could indicate increased trafficking or migration into the tissues, would be informative. In addition, transcriptional profiling of reactivated cells/tissues could reveal changes in gene expression patterns indicative of cellular activation, trafficking, or other biological processes associated with the increase in genome detection. Finally, differences between mice and humans in the growth factors and cytokines required for hematopoietic development may impact HCMV’s ability to modulate differentiation and reactivation. Nevertheless, ongoing advancements in humanized mouse models for the study of a number of human infectious diseases (142) will continue to improve our understanding of how HCMV interacts with human tissues.

## MCMV MEMORY INFLATION—WHAT DOES IT TELL US ABOUT LATENT GENE EXPRESSION?

MCMV (recently reclassified as a muromegalovirus) infection of mice has several advantages as an animal model for studying CMV infections *in vivo*: the virus is host-specific; mice are an inexpensive and accessible resource; and genetically modified animals can be used to address the role of specific cell types or cellular factors. MCMV establishes latency in endothelial and mesenchymal cells, while hematopoietic cells contain only a minority of latent genomes, and other sites of viral latency may also exist (143–148). Remarkably, although MCMV shares many direct homologs of HCMV genes, it lacks most of the unique short (US) region as well as the unique long (UL)*b′* region (149). Multiple important determinants of HCMV latency are encoded in both the US and UL*b*′ regions—including US28, UL135, UL136, and UL138—yet MCMV manages to establish a functionally similar latent state without these genetic loci or clear orthologs (29, 149–151). Furthermore, the major immediate early promoter region (MIEP) of MCMV differs notably from the HCMV MIEP, lacking important transcription factor binding sites (71, 152, 153). Transcriptional activity at the MIEP is crucial for reactivation from latency, and the differences in this locus suggest that MCMV may respond to different host cues for reactivation.

During latency, MCMV induces a unique T-cell memory response, distinguished from normal memory responses by a gradual increase in oligoclonal effector CD8^+^ T cells in a process known as “memory inflation” (154). This phenomenon also occurs in humans during infection with HCMV, although the allelic diversity of humans means that “inflationary” T-cell epitopes are less conserved (155, 156). Inflationary T cells are highly cytotoxic, retain full effector potential and all the hallmarks of recent antigen exposure, and may represent 20% or more of the total CD8^+^ T-cell population in infected hosts (154, 157–160). Inflationary T cells in mice recognize a variety of immediate-early and early-phase proteins, and in humans often recognize peptides from pp65, an early- to late-phase structural protein (154, 155, 161). These findings suggest that even during the subclinical latent state and in the absence of detectable viral replication, CMVs produce sufficient structural proteins to repeatedly prime CD8^+^ T cells. Two possibilities follow, both requiring a loosened definition of latency. First, during latent infection, stochastic expression of a few viral proteins may occur due to “desilencing” of their chromatinized genomic loci. While this hypothesis has some support in MCMV (159, 162), it is difficult to demonstrate that detection of mRNA transcripts during latency represents only a single de-silenced gene rather than a focal reactivation event. The second possibility is that reactivation and replication occur in a fraction of infected cells and are rapidly eliminated by the host. This hypothesis is also somewhat supported in MCMV studies (160). These possibilities are not mutually exclusive, and both may be at play in T-cell memory inflation.

An additional factor pointing to the possibility that broad viral gene expression occurs during MCMV latency is that different mouse strains elicit responses to different T-cell epitopes. In BALB/c mice, the canonical epitopes are derived from only two proteins (IE1 and m164) (154). In C57BL/6 mice, memory inflation occurs to cognate epitopes derived from as many as four proteins (IE3, M38, m139, and M102) (163). Notably, there is only weak correlation with the temporal kinetics of gene expression (slightly favoring immediate-early rather than early- or late-phase proteins), and incomplete evidence that these genes are regularly expressed during latent MCMV infection (144, 157–159, 163, 164). The fact that 30 years of intensive study of MCMV continues to yield surprising examples of immune responses (159, 160) and novel reservoirs of latent virus (144–146) suggests that HCMV may also persist in unexpected places.

## RCMV GENE EXPRESSION PROFILES VARY DEPENDING ON TISSUE

RCMV models of disease have played an important role in advancing our understanding of the role of CMV in vascular disease development and in bone marrow and solid organ transplant rejection. Furthermore, there has been at least one report of RCMV infection of pregnant rats as a robust fetal transmission model (165). Infection of newborn rats can cause lethal disease, whereas infection of adult rats can progress through three phases: acute viremic, persistence, and clinical latency (166, 167). Acute infections are characterized by widespread infection of many rat tissues, including major organs, salivary glands, and lymph nodes for about 10 days of infection (166, 168). Viral persistence occurs in most tissues but is especially prevalent in those that promote long-term shedding of infectious virus into saliva and urine (169, 170). Eventually, clinical latency is observed with very little infectious virus produced except from the salivary glands, which are prone to low-level virus production after viral loads in other tissues are below the limit of detection. During this phase, latency is achieved in myeloid lineage cells present in the bone marrow. However, other sites of latency also exist, possibly in vascular cells or specific tissue niches. It remains to be determined if persistent viral genomes are found in tissue cells or resident hematopoietic cells. Like HCMV, latent RCMV can be reactivated in the context of solid organ transplantation, where transplantation of donor hearts collected from latently infected rats (up to 180 days post-infection) results in RCMV reactivation and transfer of infection to naïve recipient rats, promoting transplant vascular sclerosis and allograft rejection (168). While the identity of the specific cell type capable of maintaining latent virus is still unclear, the presence of large immune complexes in heart tissues from latently infected animals suggests that the virus is maintained in tissues long-term. Like in humans, immune suppression of latently infected rats promotes viral reactivation, indicating that the immune response is not clearing but is limiting replication (171).

Like HCMV, RCMV infects many different cell types in culture, and the kinetics of infection and virus production can vary dramatically. Fibroblasts support robust and rapid replication kinetics with broad gene expression, where vascular smooth muscle cells or endothelial cells, albeit productive, are generally slower and display a more limited gene expression profile (172–174). Interestingly, bone marrow-derived macrophages and dendritic cells, as well as cultured salivary gland-derived epithelial cells, have a much more limited gene expression program (175). The differing subsets of RCMV genes detected support a hierarchy of RCMV gene expression that is cell-type-specific. Viral gene expression *in vivo* also follows a tissue- and cell-type-specific gene expression program during the acute phase. Viral gene expression was broadest in the spleen and most limited in the lung and salivary glands (170). The viral gene profiles in each tissue are unique, with only a subset of genes expressed across all tissue types. Interestingly, the profile in salivary glands at 7 days post-infection is much broader with an almost *in vitro*-like lytic program that shifts by 10 dpi to a much more limited and stable profile (176). This viral gene program is also very similar to the transcriptional program observed in infected heart tissues at 7 days post-infection. However, following heart transplantation, RCMV gene expression changes to a more lytic program, suggesting that CMV gene expression responds to inflammatory environmental cues that drive viral reactivation and cellular activation required to form replication-competent cell types (176). Profiles of CMV gene expression have not been as intensively studied in other animal models, but RCMV studies indicate a cell-type-specific and highly dynamic program of gene expression that readily responds to changes in host physiology. Given the broad low-level gene expression detected in human hematopoietic cells and the smoldering, low-level infections observed in endothelial and epithelial cells, HCMV may share many characteristics of infection with RCMV.

## PRIMATE MODELS

The CMVs that infect humans, chimpanzees, and rhesus macaques (RMs) are more closely related to each other than to other rodent CMVs (177–179). Epidemiologically, rhesus CMV (RhCMV) can be transmitted between animals via saliva or urine, with seroprevalence increasing to 100% by 1 year of age (180, 181). Once infected, viral shedding occurs intermittently throughout the lifetime of the animal (182, 183). For these reasons, primate models have been important in investigating HCMV persistence, despite the associated restrictions and cost.

RhCMV infection of RMs recapitulates many aspects of HCMV infection in immunocompetent and immunosuppressed individuals (184, 185). Experimental infection of CMV-naïve animals results in initial viremia at the site of infection along with virus detection in the blood, spleen, heart, lung, kidneys, and other organs (186–189), despite no clinical symptoms. Furthermore, RhCMV-infected cells can be detected in the parotid and submandibular salivary glands, endothelial cells, and macrophages of otherwise healthy RMs (187). Patterns of viral gene expression *in vivo* demonstrate that RhCMV orthologs of HCMV UL22A, UL146, and UL147 are the most abundant transcripts found in lymph nodes, lung, and salivary glands early after infection (190). Interestingly, viral gene expression patterns from the same tissues were more similar across RMs than across different tissue samples within the same animal (190), which parallels observations with RCMV infection of rats, highlighting how different transcriptional profiles in different cell or tissue types may influence the ability to establish latency.

After primary infection, reappearance of RhCMV DNA in the plasma is used as an indicator of viral reactivation (191, 192). Immunological responses to RhCMV in RMs also closely recapitulate what has been observed in humans. Infection with SIV or treatment with immunosuppressive drugs results in overt disease, indicating that the adaptive immune response effectively limits RhCMV reactivation and/or replication in RMs (188, 193–195). By these measurements, studies have reported frequencies of RhCMV reactivation and disease in the range of 14%–89% (185, 196–198). Gene products that control reactivation of HCMV in human *in vitro* and *in vivo* model systems, including US28 (25, 57, 95, 96, 199), vIL-10 (200), UL7 (20, 22), and UL8 (33) are present in the RhCMV genome (190, 201). The UL133-138 region, important for HCMV latency and reactivation, is positionally maintained in the RhCMV genome, but the identity of functional ORFs has not yet been determined (202).

Studies aimed at using RhCMV as a vaccine vector to express antigens derived from SIV (203, 204) have provided important insights into the persistence of CMV. The 68-1 strain of RhCMV elicits high-frequency, unconventional CD8^+^ T cells that are restricted by antigens presented in the context of MHC-II and the non-polymorphic MHC-E (205). Using this model system has demonstrated that these effector memory CD8+ T-cell responses are maintained essentially for the lifetime of the animal. Like with memory inflation during MCMV infection, mechanistically, this would require persistent exposure to antigen and suggests either long-term low-level infection, relatively frequent reactivation events that stimulate effector memory CD8^+^ T cells, or stochastic expression of viral proteins recognized by these T cells.

In the interest of minimizing pathogenicity and transmissibility of vaccine vectors, RhCMV 68-1 vectors deleted for pp71 (UL82) were found to induce long-lasting unconventional CD8^+^ T-cell responses similar to 68-1 infection, and viral shedding and spread were essentially prevented (206). RhCMV-positive but vector-naïve animals receiving bone marrow and blood leukocyte transfer from 68 to 1 vector-vaccinated animals rapidly acquired SIV-specific responses, while those who received cells from animals vaccinated with pp71-deleted vectors never generated SIV-specific T-cell responses. These data have several possible interpretations: (1) the pp71-deleted vectors were never able to productively infect myeloid lineage cells or (2) the virus was incapable of producing and/or presenting antigens in myeloid cells. Despite these possibilities, animals infected with this attenuated vector-maintained effector memory T-cell responses to the SIV transgene long term, indicating that, despite a significant attenuation in replication and shedding, some antigen expression must occur in an unknown (non-circulating leukocyte/non-bone marrow) viral reservoir.

## INSIGHTS INTO VIRUS-ENCODED CONTROL OF LATENCY

The classical view of latency, first defined in the gamma herpesviruses, was a program characterized by expression of only a limited number of latency-associated genes. The alpha herpesvirus HSV-1 followed suit with latency marked by a remarkably quiet infection where transcription is largely restricted to the latency-associated transcripts (207). This paradigm influenced the early work in beta herpesvirus latency, which focused on characterizing HCMV latency-associated genes. US28 (134, 208), UL111A (209), UL138 (151), and UL81-UL82ast or latency unique natural antigen (LUNA) (210, 211) were identified in natural latency and confirmed using various experimental models for HCMV latency; however, these pro-latency genes are also expressed during replication. In recent years, large-scale sequencing studies have challenged the dogma of a transcriptionally silent HCMV latency, failing to reveal any genes specific only to latency or replicative states, and have refocused efforts in the field (5, 27, 39, 48, 49). Instead of a binary “on/off” switch for viral gene expression (212), we have begun to appreciate a more nuanced system of control consisting of a threshold for viral and/or cellular factors that need to be reached to overcome checkpoints sufficient to reactivate a replicative state. Consistent with this, the Stern-Ginossar group has shown that such as the levels of IE1/2 and intrinsic interferon-stimulated gene levels at the time of infection dictate the outcome of infection in monocytes (213). Furthermore, while many of the virus-host interactions impacting latency and reactivation are physical interactions, we are increasingly beginning to understand complex epistatic interactions where multiple viral proteins or miRNAs coordinate the regulation of host pathways to affect outcomes of infection. While this section is not meant to be comprehensive, Fig. 2 summarizes known viral factors described as regulating the lytic-latent switch, and a few examples are expanded below. Furthermore, it should be noted that this section by no means describes all cellular pathways important to latency; due to space constraints, we list those only where a viral factor has been shown to regulate the pathways with an impact on latency and reactivation.

Epidermal growth factor receptor (EGFR) is an entry receptor for HCMV infection of CD34^+^ HPCs, and signaling through this pathway contributes to the establishment and maintenance of latency (43, 97). EGFR signaling through MEK/ERK activates the cellular transcription factor early growth response protein 1 (EGR-1) to induce expression of the HCMV *UL138* latency determinant (42). *UL138* activity restricts immediate early viral gene expression (30) and sustains EGFR/EGR-1 signaling (42, 43) to promote latency. By contrast, the pro-replicative viral gene *UL135* disrupts EGFR signaling via turnover of EGFR from the cell surface (32, 43). The viral microRNA miR-US5-2 further attenuates EGFR signaling (214), and miR-US22 downregulates EGR-1 (24) to promote reactivation from latency. The extent of these interactions suggests an important model by which HCMV has evolved to sense stress indicated by changes in major host homeostatic signaling pathways and to be responsive to corresponding changes in host transcriptional activation that can drive changes in viral gene expression either to maintain a latent infection or re-enter a replicative state. Similarly, NGF/TRK1 signaling in neurons (which initiates PI3K and AKT signaling like EGFR) is important for HSV-1 latency (215). These findings also exemplify the complexity in regulating latency as multiple virus-encoded factors (e.g., proteins and miRNAs) regulate a single host pathway at multiple points and sometimes with opposing effects.

Another example of the complexity of viral and cellular regulation of latency and reactivation is that of the G protein-coupled receptor US28, which has an important role in sensing the extracellular environment and converting that information into cellular signal transduction. *US28* has reported roles in the establishment and maintenance of latency as well as in driving reactivation from latency (25, 29). US28, in coordination with EphR2A, attenuates MAPK signaling to limit replication (95) and limits expression from major immediate early region by increasing CTCF binding (93) and attenuating the expression and activity of c-fos, a subunit of the AP-1 transcription factor in monocytes (199). Furthermore, US28 downregulates interferon-inducible gene expression for latency in CD14^+^ monocytes (131) and activates the STAT3-nitrous oxide axis to reprogram differentiation for latency (216). In CD34^+^ HPCs, ligand-specific activation of US28 signaling can fine-tune hematopoietic differentiation to modulate viral replication (25), and G protein coupling and signaling through RhoA are required for viral reactivation in latently infected CD34+ HPCs and humanized mice (57, 217). Together, these findings indicate that US28 ligand-dependent signaling fine-tunes the reactivation process. The role of *US28* in determining the latent or replicative state has been studied in other models of latency and reactivation (89, 96, 199) with results suggesting that the functional outcome of US28 signaling is likely context dependent with regard to both cell type and ligand binding, a recurring theme in studies of CMV infection.

A combined approach, using primary CD34^+^ HPC and huNSG mouse models, has been instrumental in studying the role of several viral genes and miRNAs involved in latency and reactivation (13, 20, 25, 34, 57, 140, 202). The unique advantage of the huNSG model lies in its capacity to assess the physiological functions of viral genes involved in HCMV latency and reactivation, while also gaining insight into the dynamic interplay between viral genes and the host environment. For instance, *UL136* encodes at least five protein isoforms, which exhibit cell-type-specific phenotypes for HCMV replication in both CD34^+^ HPCs and endothelial cells. The 33- and 26 kDa isoforms are required for reactivation and replication, respectively, in these models, whereas the 23-/19-kDa isoforms are suppressive for replication, such that their disruption results in a highly replicative virus that cannot establish latency (20, 45, 140, 218). These phenotypes are consistent in both CD34^+^ HPCs and huNSG mice. Notably, the disruption of the 25-kDa UL136 isoform leads to a virus that is more susceptible to reactivation *in vitro* (140), but the same virus fails to reactivate in huNSG mice, indicating a potential role for this protein within the context of an intact living organism that is not yet fully understood (140).

HCMV reactivation from latency has long been inherently linked with cellular differentiation along the myeloid lineage, representing a classic chicken or egg scenario where the inciting event cannot be pinpointed with certainty. Instead, it appears that a complex interplay between virus and host drives the interlinked processes of cellular differentiation and viral reactivation. HCMV infection of CD34^+^ hematopoietic progenitors specifically alters differentiation through virus/host interactions (20, 23, 25). In addition, a number of cellular genes that are differentially expressed depending on cellular differentiation have been identified as important for modulating HCMV latency and reactivation. For example, the cellular transcription factor EGR-1 is highly expressed in hematopoietic stem cells and plays an important role in the maintenance of stemness (219). EGR-1 also drives the expression of the latency determinant *UL138 (*42), making hematopoietic progenitors more conducive to supporting a latent infection. As infected cells differentiate, decreased EGR-1 levels would be expected to result in a corresponding drop in *UL138* expression levels. The HCMV miRNA miR-US22 downregulates EGR-1 and represents a mechanism by which the virus can control CD34+ HPC proliferation/differentiation and viral reactivation (24). Similarly, the host E3 ubiquitin ligase, inducible degrader of low-density lipoprotein receptor (IDOL), targets the pro-replicative HCMV UL136 p33kDa protein for degradation during latency establishment. IDOL is highly expressed in progenitor cells, and its expression is diminished as cells differentiate down the myeloid lineage (45), presumably allowing UL136p33 levels to accumulate for reactivation. Furthermore, the forkhead box (FOXO) family of transcription factors plays a critical role in hematopoietic development by balancing self-renewal of progenitor cells with differentiation to mature hematopoietic lineages (220). During latency, viral miR-US5-1 and miR-UL112-3p reduce the abundance of FOXO3a transcripts and proteins while HCMV pUL7 signals through the FLT3 receptor to inactivate FOXO3a by inducing its phosphorylation and translocation from the nucleus to the cytoplasm (22). FOXO3a expression and activity increase with myeloid differentiation and drive transcription from the major immediate early intronic promoters to synthesize high levels of the viral transactivators IE1 and IE2 for reactivation from latency (44). In addition, the human miR-200 family of microRNAs is more highly expressed in CD34^+^ HPCs and monocytes compared to terminally differentiated macrophages and target HCMV *UL122* RNAs, resulting in suppression of IE2.

Viral and cellular miRNAs that are differentially expressed during latent versus replicative infection likely play important roles in modulating latency and reactivation (51, 221–223). For example, HCMV miRNAs miR-US25-1-5p (224) and miR-UL148D (26, 225) target various cellular genes, resulting in decreased viral replication, which may promote latency or delay viral reactivation. In addition, HCMV miR-UL112-1 downregulates viral immediate early genes (226) and HCMV miR-US33-5p targets UL16 (227), both of which result in decreased viral replication. By contrast, HCMV miR-UL36 downregulates the latency determinant UL138 (228), which would presumably promote increased viral replication and reactivation from latency. These examples, while not comprehensive, paint a picture whereby HCMV has evolved to use or be regulated by host biology to quiet its infection in latency, and changes in that biology, many tied to myeloid differentiation, can then trigger reactivation events. The degree to which the biology of HCMV and its host are intertwined is remarkable and will require varied and overlapping model systems and innovation to functionally parse out.

## CLOSING REMARKS

A codified definition of HCMV latency remains elusive, but it is clear that CMV latency is borne out of the sum of a multitude of subtle pushes and pulls that strike a delicate balance with host biology. Work over the past two decades has shown deep integration of the virus into host biology through its interactions that allow the virus to sense and respond to changes in cellular state to enter, maintain, or exit the latent state. There are likely diverse reservoirs for latent virus, and it will be important to understand the biology of infection in these cell types and the long-term residence of latently infected cells in tissues. It will also be important to understand the contributions of latently infected cell reservoirs to persistence and the impact on the immune repertoire. Lifetime persistence of the virus and the relative maintenance of a latent state versus chronic intermittent shedding have important implications for immune activation and expansion, as well as the potential to impact chronic diseases and other infections (71, 229). It is important to continue to understand how HCMV infection programs are controlled by host pathways and how the virus senses and responds to stress, inflammatory, or environmental cues that drive viral reactivation and cellular activation required to form replication-competent cell types. It is becoming clear that latency in the organism is even more elusive for CMV, and perhaps all herpesviruses, as low levels of asymptomatic virus shedding appear to be more the rule than the exception as methods of detection increase in sensitivity.
